# Acoustic Transducers as Passive Cooperative Targets for Wireless Sensing of the Sub-Surface World: Challenges of Probing with Ground Penetrating RADAR

**DOI:** 10.3390/s18010246

**Published:** 2018-01-16

**Authors:** Jean-Michel Friedt, Gilles Martin, Gwenhael Goavec-Mérou, David Rabus, Sébastien Alzuaga, Lilia Arapan, Marianne Sagnard, Émile Carry

**Affiliations:** 1 Franche-Comté Électronique, Mécanique, Thermique et Optique—Sciences et Technologies (FEMTO-ST), 15B Avenue des Montboucons, 25000 Besançon, France; gilles.martin@femto-st.fr (G.M.); gwenhael.goavec@femto-st.fr (G.G.-M.); emile.carry@femto-st.fr (É.C.); 2 SENSeOR SAS, 505 route des Lucioles, 06560 Valbonne, France; david.rabus@femto-st.fr (D.R.); sebastien.alzuaga@femto-st.fr (S.A.); lilia.arapan@femto-st.fr (L.A.); 3 Frec|n|sys SAS, 18 Rue Alain Savary, 25000 Besançon, France; marianne.sagnard@frecnsys.fr

**Keywords:** acoustic transducer, surface acoustic wave, cooperative target, wireless sensing, ground-penetrating RADAR

## Abstract

Passive wireless transducers are used as sensors, probed by a RADAR system. A simple way to separate the returning signal from the clutter is to delay the response, so that the clutter decays before the echoes are received. This can be achieved by introducing a fixed delay in the sensor design. Acoustic wave transducers are ideally suited as cooperative targets for passive, wireless sensing. The incoming electromagnetic pulse is converted into an acoustic wave, propagated on the sensor substrate surface, and reflected as an electromagnetic echo. According to a known law, the acoustic wave propagation velocity depends on the physical quantity under investigation, which is then measured as an echo delay. Both conversions between electromagnetic and acoustic waves are based on the piezoelectric property of the substrate of which the sensor is made. Investigating underground sensing, we address the problems of using GPR (Ground-Penetrating RADAR) for probing cooperative targets. The GPR is a good candidate for this application because it provides an electromagnetic source and receiver, as well as echo recording tools. Instead of designing dedicated electronics, we choose a commercially available, reliable and rugged instrument. The measurement range depends on parameters like antenna radiation pattern, radio spectrum matching between GPR and the target, antenna-sensor impedance matching and the transfer function of the target. We demonstrate measurements at depths ranging from centimeters to circa 1 m in a sandbox. In our application, clutter rejection requires delays between the emitted pulse and echoes to be longer than in the regular use of the GPR for geophysical measurements. This delay, and the accuracy needed for sensing, challenge the GPR internal time base. In the GPR units we used, the drift turns out to be incompatible with the targeted application. The available documentation of other models and brands suggests that this is a rather general limitation. We solved the problem by replacing the analog ramp generator defining the time base with a fully digital solution, whose time accuracy and stability relies on a quartz oscillator. The resulting stability is acceptable for sub-surface cooperative sensor measurement.

## 1. Introduction: Acoustic Wave Transducers as Passive Cooperative Targets

When using classical acoustic (seismics) and electromagnetic geophysical prospection techniques, measurements currently focus on monitoring sub-surface interfaces such as voids [1], pollution plumes [2], water table/permafrost depth [3,4] or moisture concentration [5]. In the context of civil engineering and building integrity assessment, rebar or concrete homogeneity are classically investigated [6] with similar techniques, but operating at higher frequencies to improve spatial resolution at the expense of shallower depths. In this paper, we describe a cooperative target architecture enabling the use of existing hardware—GPR (Ground-Penetrating RADAR)—to complement sub-surface measurements with probing physical quantities including stress [7], temperature [8], pressure [9], torque [10] or chemical compound [11] concentration. As the sensor is embedded in concrete or buried in soil, no battery replacement can be considered after installation: the sensing element is passive, with no local battery source, and acts as a cooperative target to the RADAR wave, backscattering the information relative to the measured quantity. In our experiments, the information is conveyed through the reflected echo delays. Applications aim at assessing pavement, building or civil engineering aging conditions or integrity when subject to natural hazards such as earthquake [12], tsunami [13,14] or typhoons [15] in the post-event inspection, beyond remote sensing techniques [16] or active sensor networks [17].

Well before the advent of RFID (RadioFrequency IDentification) tags for wireless radiofrequency communication between an active reader electronic system and a passive transducer, the idea of designing RADAR cooperative targets with varying properties was imagined from the experience acquired in monitoring reflected electromagnetic wave characteristics during the Second World War [18].

RFID devices are commercially successful even though they are based on a semiconductor system [19], which must be powered by a DC signal generated by the rectification of the incoming AC signal whose amplitude must exceed the diode threshold voltage. By contrast, cooperative targets following linear energy conversion processes [20] provide several benefits:

- always returning a signal however minute the incoming power
- resistance to harsh environments
- simple design and manufacturing, often with a single lithography step, as well as operating principles.

Dielectric cooperative targets are plagued by large dimensions: delaying the signal returned by the target beyond clutter requires a dielectric delay line at least as long as the longest travel path of the electromagnetic wave. Such a geometrical constraint is incompatible with compact sensors, with the option of virtually extending the dimensions of the transducer by multiplying the number of times the wave travels along the path (dielectric resonator approach). It is to be noted that the information qualified here as clutter, or noise and artifacts in the returned signal that prevent detecting the echoes, is the same information that would be called interface reflections in classical GPR investigations. Furthermore, the sensing principle is restricted to varying intrinsic properties of the dielectric material the target is made of—dielectric dependence with temperature or moisture—frustrating the surrounding electromagnetic field or feeding the delay line with a varying load acting as the sensing element itself [21,22,23].

Since the early days of analog radiofrequency signal processing, the conversion of electromagnetic waves to the $10^{5}$ times slower acoustic wave was identified as a means of shrinking the transducer dimensions. At 300 MHz, a 1-m wavelength electromagnetic transducer is shrunk to a 10 µm wavelength acoustic transducer, meeting the compact sensor design requirements. Furthermore, using a piezoelectric substrate for converting the electromagnetic to acoustic waves offers the opportunity of making the best out of the anisotropy of the substrate: varying the acoustic wave propagation direction allows for varying dependence of the acoustic velocity (and hence returned signal delay) with environmental conditions.

Various characteristics of a cooperative target property can be investigated: RADAR cross-section (i.e., returned magnitude), resonance frequency, echo time of flight, quality factor, etc. The former is a poor selection due to being excessively sensitive to the link budget and antenna impedance on both ends of the communication channel. Frequency, along with its reciprocal time, is the physical quantity measured with the highest accuracy and reproduced most easily with excellent references available in compact and low power formats. Practically, the time delay of a periodic signal is measured as a phase and hence as the integral of the frequency. Selecting the measured quantity is not solely a matter of transducer design; both (wideband, high timing resolution) delay line and (narrowband, high frequency resolution) resonators are readily designed using classical acoustic transducer geometries. Selecting the quantity under investigation and hence the sensor architecture is a matter of the systems engineering approach, including interrogation system design, intended application conditions, possibly existing hardware and, not least, compliance with regulations if commercial applications beyond the laboratory investigation are considered.

The paper is organized as follows: first, we briefly review the surface acoustic wave transducer architecture, focusing on the reflective delay line we will be using throughout these experiments. The suitability of this sensor architecture to the pulsed mode GPR electronics is then developed, with an emphasis on the antenna characteristics’ dependence on the environment and how RADAR and sensor spectral characteristics must match. The wireless interrogation of the passive transducer acting as a cooperative target sensor is then demonstrated in a relevant environment with a measurement range well beyond 1 m when the sensor is buried in homogeneous gravel or sand. GPR timebase drift is demonstrated as the factor limiting the detection limit, and the source of this drift is identified as being caused by the stroboscopic timebase generator. A digital electronics solution to this drift issue is demonstrated, in a setup suitable to modify existing commercial GPR units in order to meet stability requirements for sensor interrogation.

## 2. Acoustic Sensor Architecture

Surface acoustic wave transducers are classical analog radiofrequency processing elements widely described in the literature [24,25,26]. Their use as wireless passive sensors goes back more than 30 years [27,28,29,30], and yet, wide acceptance remains elusive [31,32], in the context of wireless, passive sensors, possibly due to the challenges of deploying the wireless RADAR system needed to probe their response. Targeting an audience of RADAR users already familiar with the challenges of radiofrequency wave propagation and antenna deployment might leverage this limitation. Such an audience is however hardly familiar with the details of acoustic transducers, which will be described here.

Acoustic transducers are based on the principle of converting an electromagnetic wave into an acoustic wave through the inverse piezoelectric effect: interdigitated electrodes are patterned on a piezoelectric substrate so that the incoming electromagnetic wave induces an alternating electric field in the substrate. This alternating electric field induces a periodic motion of the crystalline lattice propagating in the piezoelectric substrate: an acoustic wave. Amongst the various wave polarizations meeting boundary conditions, surface waves are confined to the piezoelectric substrate surface and are prevented from propagating towards the bulk of the substrate. One classical polarization exhibiting an out-of plane component is the Rayleigh wave, a strongly coupled wave in lithium niobate orientation YXl/128°, exhibiting little propagation losses at sub-GHz frequencies: such a wave is hence well suited for wireless measurement.

**Figure 1:**
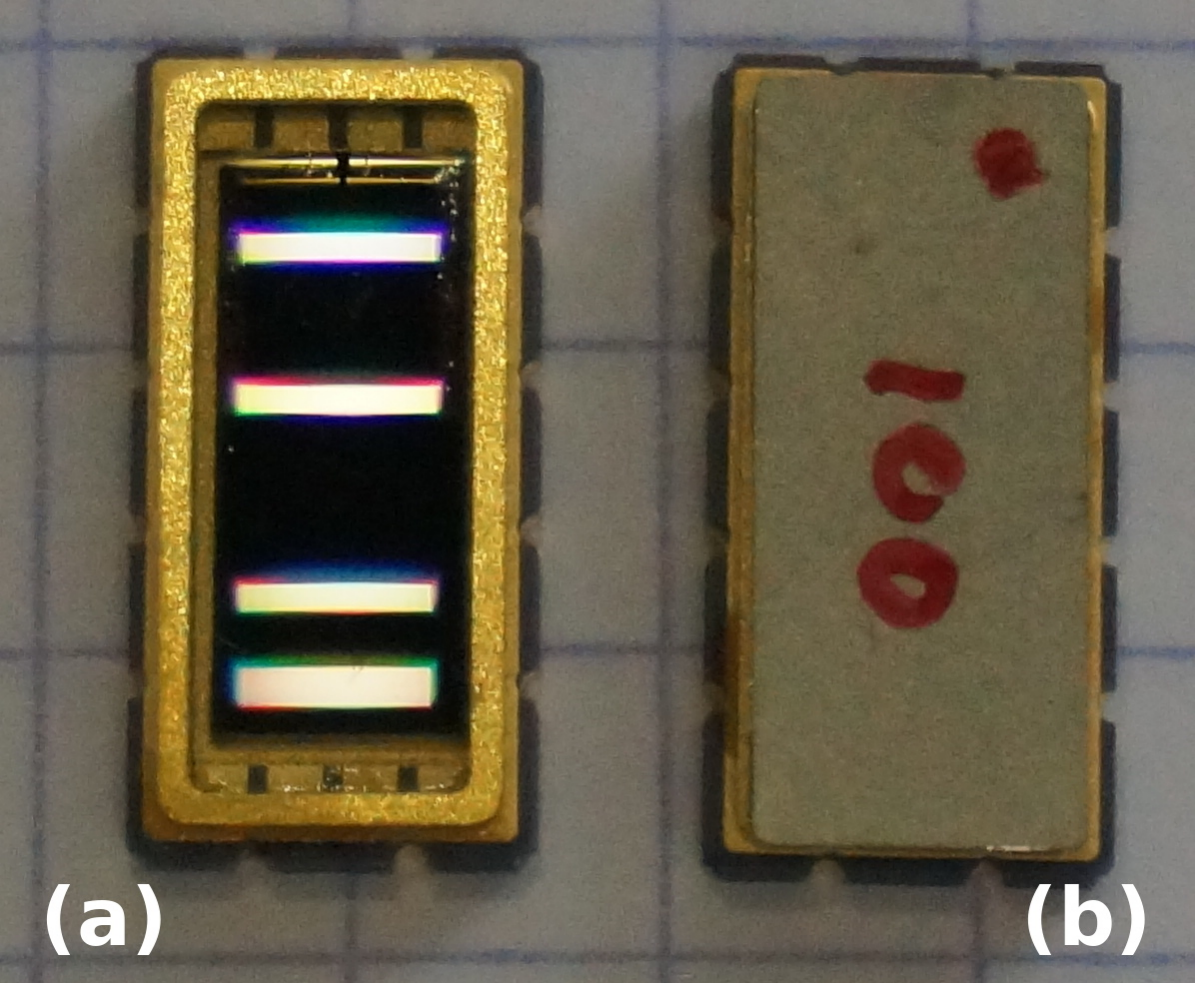
Packaged (**a**) and sealed (**b**) 100-MHz delay line made of black lithium niobate. The four light lines on the left device are reflections from the interdigitated electrode transducer (second from top) and acoustic reflectors (top and the two lines at the bottom). The grating lines on the paper used as background are separated by 5 mm.

The reflective delay line sensor architecture is based on two elements: first, an interdigitated transducer is connected to the antenna converting the electromagnetic wave into an acoustic wave by polarizing the piezoelectric substrate. The second element is a set of acoustic reflectors: the acoustic wave propagates on the surface of the piezoelectric substrate until it meets varying boundary conditions inducing acoustic velocity variations and hence reflection. In our case, electrodes patterned on the substrate induce free space to metalized boundary conditions, velocity variations and, hence, reflection of a fraction of the acoustic energy. By patterning multiple electrodes at a half-wavelength period, a Bragg reflector is designed to coherently reflect energy, hence providing a global reflection coefficient tuned by selecting the number of electrodes in the reflector (Figure 1). The reflected wave reaches the interdigitated transducer to be converted back to an electromagnetic wave through the direct piezoelectric effect. From the user perspective, the surface acoustic wave transducer acts as an electric dipole delaying the incoming wave by a few hundreds of nanoseconds to a few microseconds, with sub-centimeter dimensions thanks to the slow acoustic wave propagation velocity.

Acoustic wave propagation is sensitive to the environmental properties of the piezoelectric substrate. Temperature affects the elastic constant and the density of the substrate through dilatation: acoustic velocity being the square root of the ratio of these two quantities, it exhibits some dependence on temperature and so does the reflected echo time of flight. Similarly, stress induces variations in the elastic properties and the propagation path length, again inducing variations in the reflected echoes’ time of flight. Enhancing one measurement property and rejecting unwanted interferences from other parameters is achieved by designing the packaging, selecting the proper piezoelectric substrate, as well as the proper crystalline orientation. The piezoelectric substrate is selected for its compatibility with the environmental conditions (e.g., Curie and twinning temperatures), while the propagation direction is selected for the wave polarization to comply with boundary conditions (e.g., liquid loading). For chemical sensing, coating the sensor surface with a compound whose acoustic velocity varies upon exposure to a chemical species will also change boundary conditions and consequently reflect the echo time of flight. Since the Rayleigh wave exhibits an out of plane component radiating in fluids as a strongly coupled propagative wave, it is only compatible with sensing chemical compounds in the gas phase: measuring in the liquid phase requires a pure shear polarization of the acoustic wave, classically found in shear waves confined on the surface by a guiding layer in a Love mode configuration [33].

The sensor architecture investigated throughout this paper is made of black (pyroelectricity free) lithium niobate in a YXl/128° cut, propagating a Rayleigh wave. The sensing demonstration will focus on temperature and gas phase chemical detection: the aim of this paper is not so much to demonstrate the well-known sensing characteristics of these devices, but to emphasize the systems approach to wireless sensing, since the RADAR electronics must exhibit a “much better” stability than the variable measured to detect the quantity under investigation. The “much better” qualifier defines the detection limit for the measurement.

## 3. Using a Ground-Penetrating RADAR to Probe Acoustic Transducers

Amongst the various electromagnetic pulse emitters—namely RADAR systems and, of interest to us here, the subset of short-range RADAR systems [34]—GPR is widely used for geophysical shallow sub-surface monitoring. Using GPR for probing acoustic transducers [35] meets two complementary requirements:

- Usage case: Probing a transducer using a RADAR system, with a link budget decaying as the fourth power of the distance, requires some technical skill of the operator experienced in the basics of antenna configurations and radiofrequency device tuning. If active sensors meet the requirements of the user, the link budget decaying as the square of the distance to the receiver is always more favorable than the RADAR link budget, making the active sensor installation easier. Therefore, passive sensors will best meet situations in which active sensors cannot be considered due to lifetime expectancy or environmental conditions. Buried sensors meet such a requirement: since the civil engineering structures in which such passive sensors can be embedded, with n anticipated life expectancy of several decades solely limited by the packaging of the sensor, cannot be met by compact, battery-powered sensors. Obviously, opening a hole in a wall to replace a battery is not an option, nor is battery leakage in concrete once the energy source is exhausted. However, introducing a foreign object, the sensor and its antenna, in the concrete structure might generate sources of possible failure with the risk of initiating accelerated aging, as is seen at the interface between concrete and rebar [36]. Packaging should be considered not only from the perspective of sensor resistance to the environmental conditions, but also with respect to its chemical and physical impact on the surrounding concrete and, hence, safety coefficient of the host (e.g., building).
- Regulation compliance: GPR are included in the class of ultra-wideband emitters (e.g., European Standard EN 302 066-1 and -2). While RFID has benefited from very favorable radiofrequency regulations with dedicated bands allowing for strong emitted power (125 kHz, 13.56 MHz, 868 MHz with maximum power reaching several watts), such bands do not meet the requirement of passive linear targets such as acoustic transducers. The latter devices must therefore comply with existing regulations designed for different purposes. GPR allows for emitting strong pulses with a bandwidth compatible with typical delay line designs and at low enough frequencies (50 to 800 MHz) to, on the one hand, penetrate deep in soil and concrete, while allowing for coarse optical cleanroom lithography with acoustic wavelengths of several micrometers.

What are the limitations of this approach considering all these advantages in using GPR for probing acoustic transducers designed for acting as cooperative targets [37]? We have identified two main limitations derived from using commercial, off-the-shelf GPR instruments beyond their intended usage: fixed wavelength rather than fixed frequency operation for most commercial GPR and a poor reference time-base insufficient with respect to the targeted accuracy.

**Figure 2:**
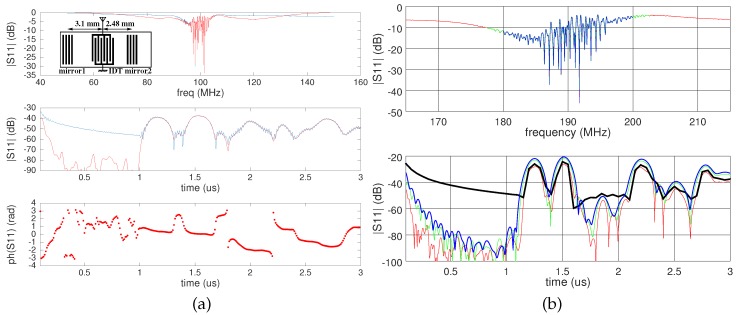
(**a**) One hundred megahertz lithium niobate reflective SAW (Surface Acoustic Wave) delay line transfer function, in the frequency domain (top: raw data in blue, windowed data in red) and the time domain (middle and bottom, magnitude and phase, respectively) as found by inverse Fourier transform of the former. Windowing (red: Blackman window) applied on the top and middle graphs (**a**) helps lower the baseline level with no effect on the insertion losses or the phase observed in the time domain. Inset: geometry of the delay line, defining the schematic position of the IDT (InterDigitated Transducer) between the two reflectors patterned on the piezoelectric substrate. (**b**) One hundred ninety megahertz lithium niobate reflective SAW delay line characterized with various bandwidths (top, frequency domain), yielding different insertion losses and time resolution (bottom), emphasizing the need to tune the probe pulse spectral characteristics to match the SAW delay line transfer function. The thick black line is the result of the inverse Fourier transform of the blue (20 MHz bandwidth) measurement, with no windowing.

All experiments are performed on reflective delay lines (Figure 2) made of 200-nm platinum electrodes patterned over a thin chromium adhesion layer. The piezoelectric substrate is lithium niobate YXl/128°, selected for its strong electromechanical coupling and high temperature sensitivity, well suited for a temperature sensor. The SAW transducers, with the electrode period tuned for the central operating frequency to be either 100 or 190 MHz, are packaged (Figure 1) in radiofrequency-compatible ceramic packages (Kyocera, Japan). The central frequencies have been selected for the SAW cooperative target to work best with 100-MHz unshielded antennas, 200-MHz unshielded antennas or 250-MHz shielded antennas, as provided by Malå Geoscience (now Guideline Geo, Malå, Sweden). All measurements are performed using the commercially available ProEx GPR control unit, provided by the same supplier. The custom control software available at [38].

Initial SAW delay line characterization is performed in the frequency domain using a network analyzer (Figure 2a,b top), assessing whether the SAW transducer response matches the GPR emitted pulse spectra. Frequency to time domain processing is needed to assess the expected GPR response, with echos delayed by durations defined by the IDT (InterDigitated Transducer) to reflector distance *d* and the acoustic velocity *v*. Since the distance is fixed and the velocity is dependent on the SAW transducer environment, the delay τ=d/v is representative of the physical quantity under investigation. Two echoes induced by two reflectors patterned on both sides of the IDT are visible in Figure 2a (middle) and Figure 2b (bottom), with delays of about 1.25 and 1.55 µs, as defined by distances *d* of 2480 and 3100 µm, respectively, consistent with the Rayleigh wave tabulated velocity of 3979 m/s [24] on lithium niobate YXl/128°. The electromechanical coefficient:

(1) $$K^{2}=2\frac{\Delta v}{v}=5.4\%$$

with Δv the metalized to free surface acoustic wave velocity difference, hints at insertion losses of $10\log_{10}\left(K^{2}\right)=-13$ dB: the higher losses (−40 dB) seen in Figure 2a are due to the excessive bandwidth of the measurement on the network analyzer whose frequency span was widened to clearly show the acoustic bandwidth of the device. The measurement bandwidth in Figure 2b was adjusted to optimize returning power by matching the probe signal bandwidth with the SAW reflective delay line bandwidth. The time delay of the echoes is accurately measured as a phase:

(2) φ=2πd/λ

with λ the acoustic wavelength, as seen in Figure 2a (bottom). Keep in mind that when converting from the frequency domain to the time domain (Figure 2a, middle and bottom) using numerical processing software such as MATLAB or GNU/Octave, their convention of positioning the zero-frequency differs from the one expected from radiofrequency signal processing: in the former software, the zero-frequency is located on the left part of the chart and the sampling frequency on the right part of the chart. Radiofrequency signal processing and demodulation expect the zero-frequency to lie in the middle of the chart, with minus half the sampling frequency and half the sampling frequency lying on the left and right part of the chart, respectively. Converting the frequency measurement obtained on a network analyzer to the time domain requires converting from one convention to the other, as conveniently provided by the fftshift() function of these processing software packages. The magnitude of the time domain response is not affected by this convention issue, but the phase is erroneous if the center frequency is not brought to the center of the chart during the conversion from the frequency to time domain.

Throughout this document, time delay through phase measurement is performed in a differential approach to eliminate the additional delay depending on the RADAR to target distance: subtracting the phase of two echoes eliminates the common time of flight term and is representative solely of the acoustic velocity. As is classically known in RADAR signal processing, the matched filter is the cross-correlation: in our case, the incoming electromagnetic pulse, converted into an acoustic wave through the inverse piezoelectric effect of the substrate, is reflected twice by two reflectors located at different distances from the IDT. These two echoes are converted back into an electromagnetic wave by the direct piezoelectric effect and detected by the RADAR. Cross-correlating the signals within the two time intervals in which the echoes are known to lie provides optimal noise rejection and signal identification. Furthermore, the cross-correlation maximum position provides a fine estimate of the time delay between the two echoes: in all our processing steps, the cross-correlation maximum is detected and fitted by a polynomial function for oversampling, assuming the cross-correlation maximum is locally symmetric and approximated at the second order by a parabola. The fine phase estimate is given by the parabola fit maximum, with a time-resolution improvement with respect to the sampling period equal to the signal to noise ratio. A core aspect of the passive cooperative target with respect to RFID measurement is that the former is an analog measurement, with a measurement range that is not defined by reaching a threshold voltage to power a digital circuit, but by a signal to noise ratio deteriorating the measurement quality until it becomes no longer usable.

The link budget is determined by the ability of the electromagnetic wave to propagate in the various media between the surface emitter and the sub-surface cooperative target, the electromechanical conversion efficiency, antenna impedance and transfer function matching to the incoming electromagnetic pulse and returning signal losses through the same medium in which the electromagnetic wave propagates.

The link budget due to free space propagation of the electromagnetic power and conductivity losses in the medium is well documented and classically investigated when assessing GPR measurement range [39]. In the case of RADAR systems, with a target acting as a point like-source, the FSPL (Free Space Propagation Loss) rises with the distance to the fourth power. Indeed, power spreads first on a sphere centered on the emitter and decays with the square of the distance to the source. The target itself acts as a point-like source from which a new sphere defines the surface over which the minute returning power spreads again. The product of two quadratic power laws yields a global loss law with distance appearing as the fourth power, an unfavorable condition with respect to powered sensors whose power law only decays as the square of the distance. Considering such a link budget balance, using cooperative targets are only justified in unique operating conditions where active sensors are not applicable. The electromechanical conversion, which replaces the RADAR cross-section of a target, is addressed by selecting strongly-coupled piezoelectric substrates and acoustic wave modes meeting both sensing capability and strong conversion efficiency: from the user perspective, an acoustic wave transducer is an electrical dipole returning a fraction of the incoming power, through a linear process (i.e., whatever the incoming power, some power will be returned). We will here discuss the last design issues, which lie in impedance matching and spectrum matching.

## 4. Fixed Wavelength Versus Fixed Frequency

Most commercially available GPR emit a pulse by unloading a radiofrequency capacitor through an avalanche transistor. The broadband pulse is then filtered by the dipole antenna fitted on the avalanche transistor emitter: the transfer function of this dipole antenna, located on the ground surface for efficient coupling, is strongly influenced by the ground permittivity. The impact of the environment permittivity and conductivity on a dipole antenna property has been extensively investigated in [40] and will be further addressed below. Since the dipole length is fixed and the permittivity (and thus electromagnetic velocity) varies, the emitted central frequency varies accordingly, as does the impedance at resonance. However, the cooperative target is designed to operate at a fixed frequency range, determined by the spacing between interdigitated electrodes patterned on the piezoelectric substrate. The returning signal is the convolution of the incoming pulse with the impulse response of the acoustic transducer in the time domain, yielding an efficiency easier to grasp in the frequency domain as the product of the pulse spectrum with the acoustic wave transfer function. Any mismatch will prevent a fraction of the incoming electromagnetic pulse power from coupling with the acoustic transducer and yield a loss in measurement range. Similarly, impedance mismatch prevents a fraction of the incoming electromagnetic pulse from being converted to an acoustic wave as it is reflected by the mismatched antenna. Ideal coupling conditions are met when the antenna impedance is the complex conjugate of the SAW impedance [41,42]. Because the buried antenna impedance widely varies with surrounding permittivity, while the acoustic transducer operates in a relatively narrow frequency band with respect to the ultra-wideband GPR operating range, such a mismatch can become significant. A broadband antenna geometry, as found for example with a bow tie antenna classically used for GPR applications, will reduce the surrounding permittivity issue at the cost of poorer efficiency in the operating frequency band of the acoustic transducer.

An initial analytical investigation in which the dipole impedance is modeled as an Resistive-Inductive-Capacitive (RLC) circuit [43] exhibits geometrical and surrounding media permittivity ε dependence through the equivalent passive components value. The capacitance value is determined by the dipole half-length *l* and diameter *a* (or maximum diameter in the case of conical radiating elements) ([44], p. 305) by:

(3) $$C=\frac{\pi \varepsilon l}{\log(2l/a)-\log(2)}$$

The inductance value *L* on the other hand has no reason to depend on the permittivity and is solely defined by the permeability and geometric properties of the dipole ([44], p. 311) so that the impedance seen at resonance, when the inductive part of the circuit compensates for the capacitive part, becomes [43]:

(4) $$R=\frac{L}{C\cdot R_{r}}$$

with $R_{r}$ the radiation resistance. This impedance at resonance is inversely proportional to the permittivity of the medium surrounding the antenna through the capacitance term, a cause of varying matching condition with the sensor impedance defined solely by its acoustic design characteristics, while the resonance frequency:

(5) $$f_{R}=\frac{1}{2\pi \cdot \sqrt{LC}}$$

of the dipole varies as $1/\sqrt{\varepsilon }$. Such analytical analysis provides a first insight into the parameters affecting matching conditions between the sensor and the antenna. We wish to investigate further and confirm such issues through numerical simulations. We do so through 3D modeling by Finite Difference Time Domain (FDTD) means.

**Figure 3:**
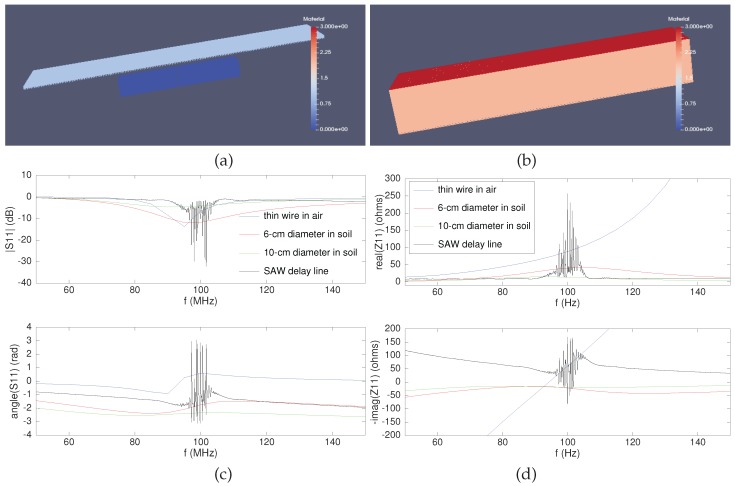
GprMax3D (www.gprmax.com) modeling of antennas using FDTD: (**a**) is a typical geometry, here of a dipole antenna with square cross-section, and (**b**) is the simulation volume excluding the perfectly-matching layer cells on the sides. $S_{11}$ (**c**) and impedance (**d**) reflection coefficients of various antenna geometries discussed in the text are exhibited, as well as the same characteristics for the SAW transducer acting as antenna load: bottom chart colors match the legend of the top charts. The soil permittivity was set to four with no imaginary part, and the dipole length halved with respect to its dimensions in air when simulated in soil. The imaginary part of the sensor impedance is displayed on the same chart as the opposite of the imaginary part of antenna impedances: matching conditions require that the complex conjugate of the antenna impedance equals the impedance of the sensor in its operation bandwidth.

FDTD modeling was selected for considering complex media surrounding the radiating element, with varying permittivity and conductivity, as well as layering or geometrical features such as pipes located near the sensor. Additionally, such modeling tools are well suited for assessing antenna geometry, bandwidth with respect to conductor diameter and impact of the various dielectric permittivities of soil and air when the sensor is buried at depths less than a wavelength. The selected implementation of FDTD is gprMax3D [45], an open source software allowing for antenna impedance characterization (Figure 3, top). A thin-wire dipole geometry exhibits a bandwidth which is too small to efficiently couple with a SAW delay line: the means of widening the operating bandwidth classically involves broadening the radiating element, so we investigated the impact of the radiating element diameter on the bandwidth and impact on the antenna impedance with the aim of meeting the SAW impedance complex conjugate matching condition (Figure 3, bottom).

Attempts at replacing the dipole with a bow tie geometry were met with simulation challenges for properly modeling the triangularly-shaped radiating element: impedance results were observed to be dependent on the mesh size of the FDTD grid, making results hardly reliable. Nevertheless, the poor efficiency of the broadband bow tie with respect to the dipole is observed, with hardly any energy coupling within the SAW bandwidth.

Since the electromagnetic wave velocity shrinks to the same extent as the square root of the medium permittivity, operating in media whose water content strongly varies—soil, sand—inducing strong permittivity variations [46], already presents a challenge of matching the antenna transfer function with the sensor transfer function. Indeed, despite being commercially defined by an operating frequency, GPR antennas actually act at constant wavelength and thus varying frequency. The surface dipole is subject to varying soil permittivities, which vary the central frequency of the broadband pulse filtered by the fixed geometry dipole whose electromagnetic length varies with permittivity. The condition is worse for the buried sensor, where again the antenna geometry is fixed, but the surrounding permittivity varies.

More reasonable than the permittivity variation of sand, concrete only exhibits minor permittivity changes [47] with moisture content, keeping the dipole transfer function within the operating band of the SAW transducer. Nevertheless, the antenna mismatch under varying environmental conditions remains an issue even if tuning the antenna properties to match the SAW transducer in a given condition.

## 5. Experimental Investigation

**Figure 4:**
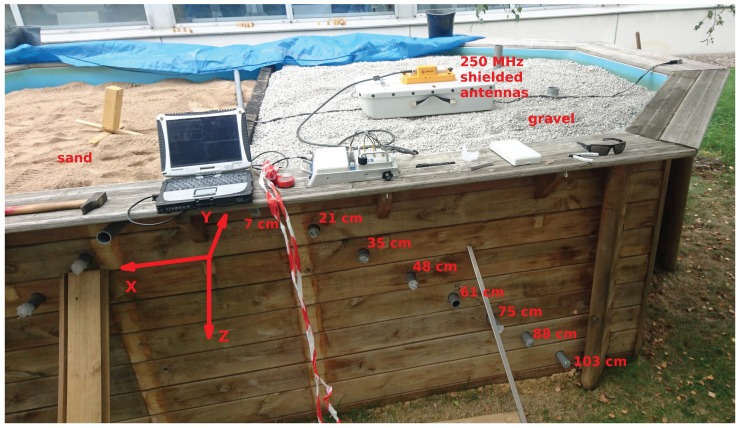
Experimental setup of the 3.3 m-wide, 5.4 m-long and 1.2 m-deep sandbox, with two media for assessing the dependence of the medium permittivity on the link budget. Fine sand (left) and gravel (right) provide environments representative of sub-surface measurement environments. The coordinate framework with Z along the depth, X along the RADAR scan direction and Y along the longest length of the antenna dipoles is used in the text to refer to the varying parameter during the measurement.

The issues of impedance matching of the sub-surface antenna with the sensor and antenna radiation pattern are addressed experimentally in a 3.3 m-wide, 5.4 m-long and 1.2 m-deep sandbox assembled specifically for these experiments (Figure 4). The fine sand (left) and gravel (right) were selected as homogeneous, well-characterized media: sensors are inserted through 40-mm diameter Poly(Vinyl Chloride) (PVC) pipes positioned at various depths, ranging from 7 to 103 cm from the surface. During all experiments, the sensors are inserted by at least 1 m in the tubes to make sure the environment is homogeneous along the whole length of the antenna attached to the acoustic transducer.

In all recorded charts, the time axis starts at the measurement window beginning time (offset by about 1 µs), which was positioned to focus on the echoes returned by the delay line and does not include the emitted pulse. Our custom software allows for defining two time-windows, one positioned close to the emitted pulse for shallow interface echo recording (not shown here), and a second focusing on the sensor response (used here). Measurement durations typically last 850 ns to 1200 ns, or 4096 samples recorded at 4556 to 3417 MHz.

### 5.1. Spatial Dependence of the Returning Signal

**Figure 5:**
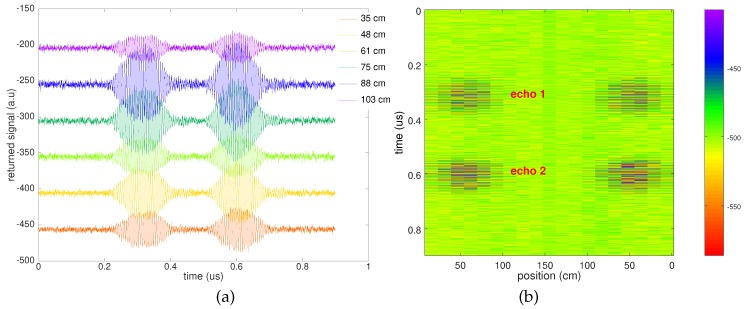
Experimental measurement of returned echoes as a function of depth (Z) of the sensor below the antennas (**a**), and as a function of antenna position along the scan axis (X) in a two-way pattern (positions 0 to 150 are right to left and positions 150 to 0 are left to right as seen in Figure 4). The shallowest sensor (**a**) exhibits a lower returning power due to its position forward from the antenna and not directly below the radiation pattern. (**b**) is a radargram recorded with the sensor buried 61 cm in gravel.

Returning signal as a function of depth is shown in Figure 5a: the signal returned by the sensor is clearly visible for all tested depths from 103 to 35 cm. The shallowest sensor position yields a poor signal due to the strong directivity of the GPR radiation pattern, with the dipole antenna preferentially radiating towards the high permittivity soil. Since the shallowest sensor position is located in front of the antenna rather than below, the antenna gain is poor in this direction, accounting for the lower signal level.

To address the antenna directivity, the GPR antenna is moved on the surface in a direction orthogonal to the tubes: the resulting radargram is exhibited in Figure 5b, with the fast time axis in the vertical range and the GPR antenna position as the abscissa. During this experiment, the sensor is kept at a fixed location and depth of 61 cm: a usable signal is only recorded as the GPR antennas are located above the sensor, with the signal attenuated below noise level at position 0 (left and right part of the chart) and abscissa between 100 and 150 cm. The distance along which the sensor signal is usable is about 1.5-times its depth, so an angular field of view of $2\times \arctan\left(0.75\right)≃70^{\circ }$ is estimated. Indeed, the high permittivity soil (with respect to air) directs the electromagnetic wave preferentially towards the higher capacitor and, hence, shapes the radiation pattern of the dipole located over the ground towards the sensor [48].

**Figure 6:**
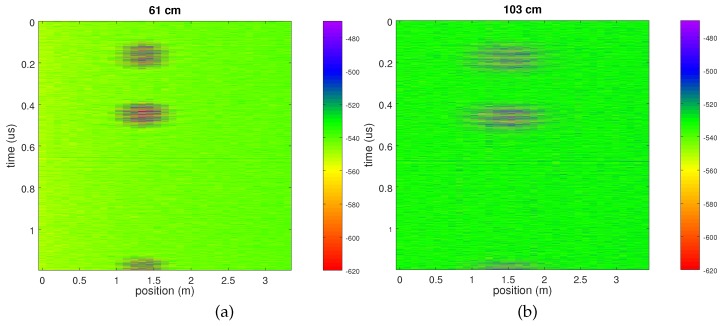
Experimental measurement of returning echoes as a function of the position of the sensor along the pipe (Y), moving in a direction parallel to the length of the dipole. (**a**) for a sensor buried 61 cm in gravel, (**b**) for a sensor buried 103 cm in gravel.

Similarly, the sensor position offset along the antenna dipole axis impacts the returned signal level. Figure 6 exhibits the results of two measurements while moving the sensor inside the pipes, parallel to the longest direction of the dipole of the GPR antenna. The length along which the sensor signal is usable is about equal to its depth, suggesting an angular field of view of $2\times \arctan\left(0.5\right)≃50^{\circ }$ of the dipole antenna.

### 5.2. Returning Signal Dependance with Antenna Dimensions

**Figure 7:**
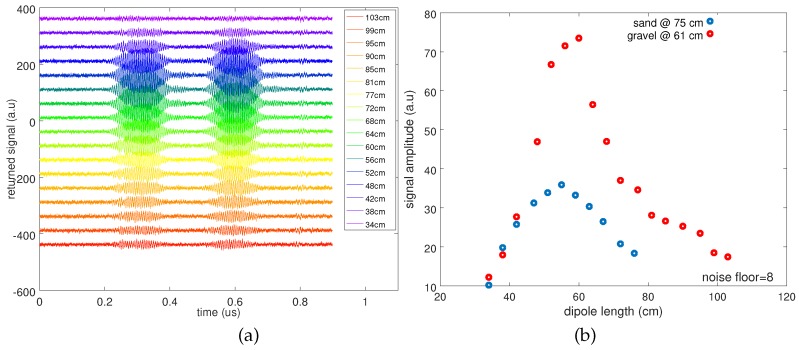
(**a**) Returned signal level as a function of dipole length, for a sensor buried 61 cm deep in gravel. (**b**) Evolution of the signal amplitude as a function of dipole length, for a sensor buried under gravel and a sensor buried in sand.

The most dramatic issue when burying the sensor is the impact of the variable permittivity of the surrounding environment on the operating frequency of the fixed-length dipole antenna 2l. Figure 7 summarizes the impact of varying dipole length on the returned signal level depending on the surrounding medium. This experiment simulates the varying permittivity of the environment since the electromagnetic velocity as seen by the dipole antenna decreases to the same extent as the square root of the surrounding media permittivity:

(6) $$\sqrt{\varepsilon }=\frac{c_{0}}{4l\times f}$$

with $c_{0}=300$ m/µs the electromagnetic velocity in free space and *f* the central frequency of the pulse, here 190 MHz. The sensor buried in gravel exhibits an optimal antenna length equal to 60 cm (two radiating elements of l=30 cm long each), suggesting a relative permittivity of $\varepsilon_{gravel}≃1.7$, close to the value tabulated in [46]. Sand exhibits a slightly higher value since the optimum dipole length is observed to be 2l=55 cm, suggesting a relative permittivity of the medium of $\varepsilon_{sand}≃2$.

This result illustrates the challenge of designing a single antenna geometry compatible with various sub-surface environments, and most significantly with varying moisture content and the associated strong variation in permittivity and, hence, of electrical length of the dipole. While the varying link budget will not prevent the measurement thanks to the fine analysis of echo delays rather than returned magnitude as observed through the RADAR cross-section, the deteriorated signal to noise ratio on the analog radiofrequency measurement will degrade the measured physical quantity signal to noise ratio. Indeed, both quantities are related by the signal processing procedure. While the sensor intrinsic characteristics do not require ultra-wideband antenna design, keeping an acceptable link budget in varying surrounding permittivity conditions requires broadening the antenna operating frequency range in order to keep an acceptable efficiency at the fixed SAW sensor operating frequency band.

Assuming the signal has been coupled to the cooperative target, which then returns echoes whose delays are representative of the acoustic velocity, i.e., physical quantity, the remaining issue is whether the time delay can be measured accurately enough to identify the acoustic sensor properties, rather than the measuring instrument (GPR receiver) drift.

## 6. Local Oscillator Stability

The second issue we raise when using a commercial GPR for probing an acoustic transducer is the local timebase stability. Piezoelectric-based transducers are classically used as frequency selective components to design oscillators: in such a case, the resonator is designed to exhibit a frequency as stable as possible as the environmental conditions are varying. For example, design techniques include selecting locally temperature insensitive piezoelectric substrate cuts and wave modes, as well as a packaging preventing stress transfer or pressure variation that might significantly affect the acoustic wave velocity and, hence, the phase, yielding varying Barkhausen conditions in the oscillator and, thus, the output frequency. When designing a sensor, the opposite design considerations are applied: the acoustic velocity is expected to vary significantly with one given quantity, while the packaging aims at preventing other physical parameters from significantly affecting the acoustic velocity. Nevertheless, even when selecting strongly dependent material cuts and wave mode to quantities under investigation, the stability of single crystal substrates rarely yields sensitivities above the hundreds of ppm (part per million) range. Thus, the timebase reference should drift by a minute amount with respect to these expected variations for the measurement to be stable and hardly affected by a baseline drift, which cannot be attributed to the instrument or the sensor measurement.

We have reported [49] the excessive timebase drift of a commercial GPR, Malå’s ProEx. As a reminder of the core result of this past investigation, we have observed when probing a SAW delay line in a fixed environment that due to the instrument timebase drift, the time delay between echoes appeared to be shifting by 15,000 ppm (1.5%, as observed through a 4.5-ns drift for echoes separated by 300 ns). Lithium niobate YXl/128° typically exhibits a temperature sensitivity in the 70 ppm/K range: the GPR timebase drift makes such a sensor unusable in the narrow temperature range expected in sub-surface measurements, where a few ppm acoustic velocity variations are expected for sub-K temperature variations. The cause of the excessive drift is the means by which the stroboscopic timebase is generated. Indeed, with a GPR typically operating in the 100 to 2000-MHz range, sampling rates above several GHz are targeted to properly identify the pulse round trip. Few direct sampling analog to digital converters allow such large sampling rates, so that a stroboscopic approach is often adopted in which multiple measurements are repeated, under the assumption of a stationary environment during the measurement, with each new sampling delayed by N×dt, *N* the sample index and dt a fixed time interval set so that 1/dt is an equivalent sampling frequency. The stability of generating the programmable N×dt delay is of utmost importance to the stability of the sampling rate, on which all subsequent digital processing is based. In this past publication [49], the timebase drift issue was identified: we will here demonstrate a solution to this issue by replacing a core element of the GPR receiver.

**Figure 8:**
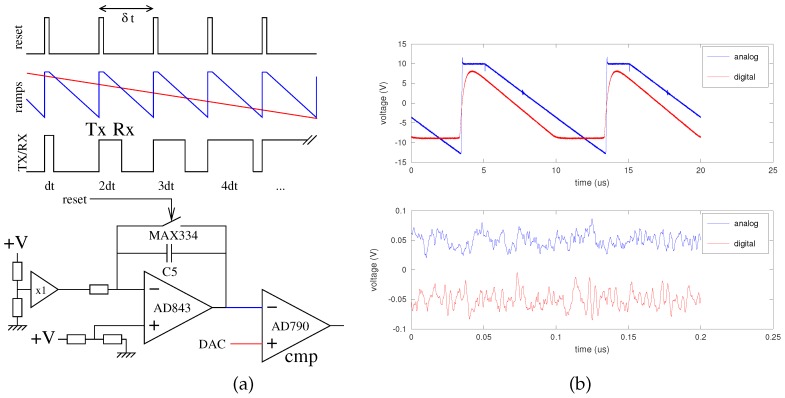
(**a**) Schematic view of the voltage to time converter for generating the stroboscopic timing signals (top), and (bottom) implementation of the fast ramp generator in the ProEx GPR. (**b**) Comparison of the analog ramp generator (blue) and our proposed digital implementation of the ramp generator (red). The slightly different slopes mean that the nominal sampling rate is no longer valid. Bottom right: zoom on the linear parts of the ramps generated by analog (blue) and digital (red) means, the latter voltage being generated by an Analog Devices AD9871 DAC: both methods show similar noise levels, as seen here after removal of the linear trend (the two curves were offset for clarity).

In Malå’s ProEx, the timebase is defined by feeding a comparator with two saw-tooth-shaped signals, as classically designed in a voltage to time converter [50]. One slowly varying saw-tooth shaped signal sweeps the voltage span of the comparator input during the measurement duration needed to collect the *N* samples (Figure 8a, top), while the second saw-tooth signal spans the whole input voltage range during the time needed to collect a single sample. The PRR (Pulse Repetition Rate) determines the largest distance at which a target can be detected and is determined in the current scheme by the ramp reset signal period. As an example, for Malå’s ProEx, the PRR is 100 kHz, so that the furthest target is located at 1500 m in air or 850 m in ice. This 10 µs maximum returned echo delay is appropriate for probing SAW delay lines with delays up to a few microseconds. Thus, collecting 1024 samples requires a stable environment between the GPR and the target during 1024×10μs≃10 ms if no additional stacking is used during acquisition. The slow ramp is generated by a digital to analog converter, a digital component whose voltage range can be defined by a stable voltage reference, and clocked by a microprocessor whose frequency is determined by a quartz oscillator exhibiting better stability than the sensor sensitivity: little drift is to be expected from this part of the circuit. The fast ramp on the other hand is generated by the integration of a constant voltage defined by a resistor bridge. The integration is performed by an analog operational amplifier circuit with a capacitor in the feedback loop, which is short-circuited by a digitally-controlled analog switch for resetting the integrator at a PRR interval (Figure 8). Capacitors are well known to be dependent on environmental conditions, whether temperature or moisture: the poor sampling rate stability of Malå’s ProEx is attributed to the fast ramp generation strategy.

In the scheme demonstrated in Figure 8, the equivalent sampling rate $f_{s}$ is given by:

(7) $$f_{s}=\frac{1}{dt}=\frac{\Delta T_{slow}}{\Delta V_{slow}}\cdot \frac{\Delta V_{fast}}{\Delta T_{fast}}\cdot \frac{1}{\delta t}$$

where $\Delta T_{slow}$ and $\Delta T_{fast}$ are respectively the slow and fast ramp period, $\Delta V_{slow}$ and $\Delta V_{fast}$ respectively the slow and fast ramp voltage amplitudes and δt the pulse repetition interval (10 µs in the case of the ProEx). As shown on Figure 8, dt is the time increment between the emitted pulse and the sampling trigger between successive pulses and is therefore the inverse of the equivalent sampling rate. This equation is useful in an error budget analysis, emphasizing the importance of the voltage ranges and most importantly the $\Delta T_{fast}$, which is the core issue in this discussion, all the other signal timing being controlled by a stable quartz-clocked oscillator.

**Figure 9:**
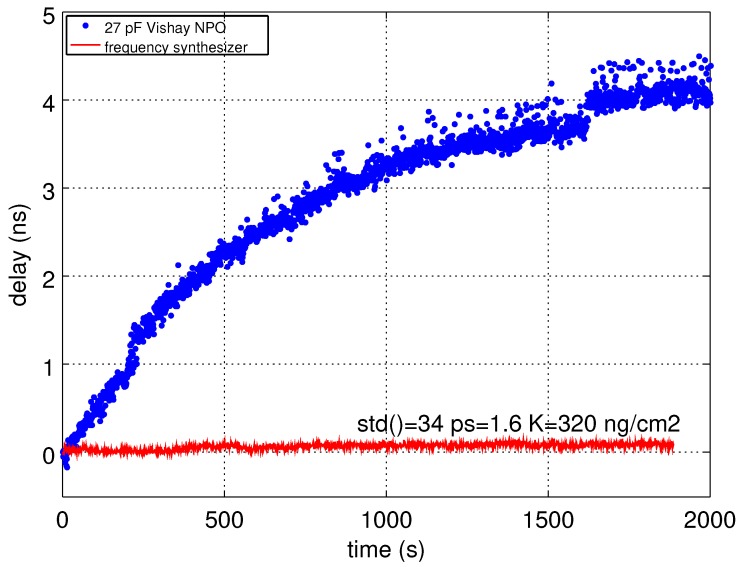
Comparison of the timing stability using the internal ramp generator provided by the receiver board in Malå’s ProEx control unit (blue) and when replacing this analog ramp generator with a Tektronix AFG3102 triggered by the reset signal provided by the GPR at the pulse repetition rate. Despite the 200 ps jitter announced in the AFG3102 documentation, the observed time delay standard deviation is close to the targeted 20 ps for 1 K temperature measurement resolution.

Targeted time delay accuracy is defined by the acoustic velocity variation induced by the physical environment under investigation of the sensor: with a 70-ppm/K temperature sensitivity, a 300-ns echo delay difference and a targeted 1 K measurement resolution, the delay measurement accuracy should be in the 20-ps range. Similarly, a 1-µg/cm^2^ adsorbed film on the surface of an acoustic transducer exhibiting a gravimetric sensitivity of 200 cm^2^/g in a chemical sensing application will exhibit a 60-ps delay upon film formation: again, a time delay accuracy of a few tens of ps should be targeted. To demonstrate this point, we replace the analog ramp generation scheme with a quartz-disciplined oscillator clocked digital ramp synthesizer. As a first demonstration, a laboratory-based experiment is performed using an off-the-shelf arbitrary waveform generator (Tektronix AFG3102) (Figure 9).

**Figure 10:**
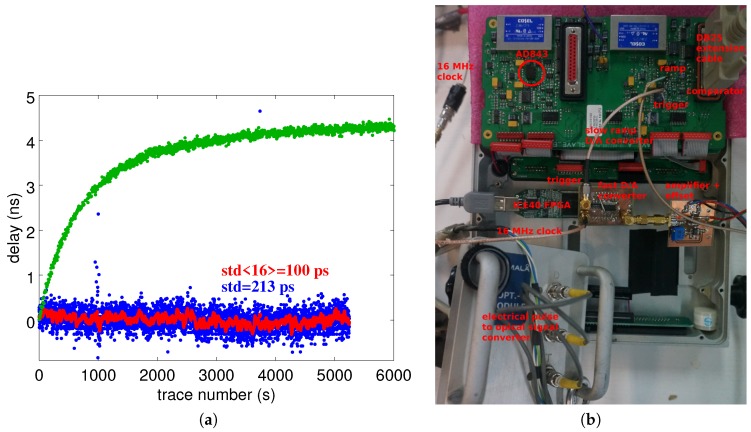
(**a**) Timing generator comparison. The time delay variation between two echoes separated by 300 ns is recorded by a ProEx GPR unit. The green curve results from the analog timing generator, the blue curve from the digital timing generator, with the digital to analog conversion performed using a discrete-component R-2R resistance network lacking clock filtering capability. The red curve results from a sliding average over 16 samples of the blue curve. All measurements are performed with 16 stack accumulation. (**b**) Experimental setup in which the faulty analog ramp generation (highlighted on the left ramp generator centered on the AD843 operational amplifier, not used during this experiment) is replaced with an FPGA-based digital ramp synthesizer.

To demonstrate the ability to transfer the concept to an embedded implementation, a FPGA (Field Programmable Gate Array) controlling a fast digital to analog converter is used. We have met three challenges in this implementation:

- An initial implementation was running on its own clock, independent of the clocks running the GPR timing circuitry. Due to the lack of synchronism between the rising edge of the reset signal defining the emitted pulse timing and the detection of this edge by the synchronous logic in the FPGA, a random delay between the emission and its detection was introduced. With an FPGA core clock of 50 MHz, such a jitter amounted up to 20 ns, an unacceptable variation with respect to the targeted sub-100 ps;
- the ProEx GPR is clocked by a 16-MHz core clock also distributed as a 1-MHz signal following division. Both clocks are readily available, and the 16-MHz clock is used to feed a direct digital synthesizer whose internal PLL (Phase Locked Loop) multiplies by 10 to reach a 160-MHz clock signal feeding the FPGA, synchronous with the reset signal. However, transmitting a 160-Hz signal to clock a circuit of a GPR operating with 100 or 200 MHz is a poor solution since the receiver stage was picking up this clock signal, which quickly overwhelmed any small echo signal that should have been recovered by the receiver;
- The final solution was the use of an FPGA both with an internal PLL and able to use an input frequency as low as 16 MHz: Lattice’s (now Microsemi) ICE40 met both conditions and was used, in conjunction with a fast digital to analog converter (AD9761), to generate the saw-tooth-shaped ramp by running a counter on the FPGA, reset by the signal that was used to trigger the analog switch. Due to excessive clock leakage, the output timing is noisier than its analog implementation, but does not exhibit significant drift over more than two-hour measurements (Figure 10).

**Figure 11:**
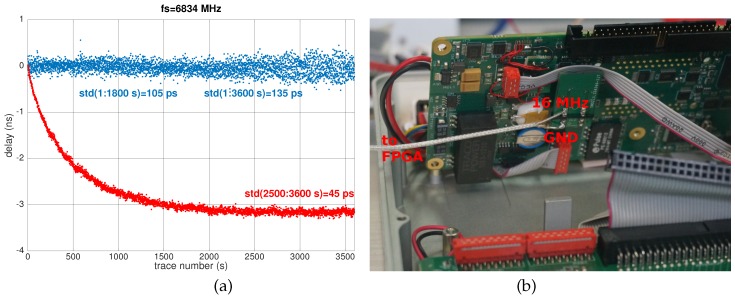
(**a**) Timing generator comparison. The time delay variation between two echoes separated by 300 ns is recorded by a ProEx GPR unit. The red curve results from the analog timing generator and the blue curve from the digital timing generator, with the digital to analog conversion performed using an AD9761 DAC. All measurements are performed with 16 stack accumulation. (**b**) The FPGA must be clocked by a reference signal synchronous with the reset pulse. The 16-MHz clock used to generate this reset pulse in the ProEx electronic circuit is derived to feed the PLL of the FPGA.

The AD9761 DAC was selected for its high speed and high resolution: sweeping the voltage range in 5 µs with 10-bit resolution requires configuring the DAC every 5000/1024 = 4.9 ns or a refresh rate of 205 MS/s. Because the clock frequency of this particular DAC is limited to 40 MS/s, we have selected to reduce the resolution to eight bits by only keeping the most significant byte of the configuration word, generate a 40-MHz clock by multiplying the 16-MHz reference clock provided by the GPR (Figure 11 b) using the PLL internal to the FPGA: thus, the 256 steps are swept at a rate of 40 MHz or a ramp duration of 6.4 µs. The DAC provides a digital low pass filter to reject the clock: the 43 clock cycle delay (lasting 1075 ns) introduced by this filter does not affect the equivalent sampling rate, but only the sampling start time, which is compensated for by tuning the voltage offset added at the DAC output.

It is to be noted that the timebase drift exhibited by the commercial instrument cannot be reliably predicted: although associated with an excessive heating of an (AD843) operational amplifier reaching temperatures above 50 °C while the GPR control unit is kept at room temperature, heating thus the surrounding passive components, as well, the observed timebase drift is either positive (Figure 10a, green curve) or negative (Figure 11a, red curve), depending on the electronic board. The drift is always the same for a given board, but the sign and magnitude of the drift vary from board to board (tested on three Malå ProEx receiver electronic boards).

Using the updated hardware in which the analog timing generator is replaced by our digital implementation, a long-term stability suitable for sensor measurement is achieved. The short-term degraded standard deviation of 100 to 130 ps reduces the measurement resolution to 5 K for a temperature sensing application of to 400 ng/cm^2^ for a chemical compound detection application. Since long-term drift has been eliminated and only random fluctuations are observed, the analog measurement standard deviation can be reduced by further averaging to reach the targeted detection limit, yielding an integration time only limited by the measurement refresh rate needed by the application or the kinetic.

## 7. Conclusions

Sub-surface deployment of passive wireless sensors probed from the surface by using a GPR (Ground Penetrating RADAR) provides a compelling scenario meeting user expectations and radiofrequency emission compliance. However, using GPR beyond its expected design reaches the limitation of the timebase stability with a local sampling rate drift well above the sensor delay variation with the sensed environment conditions. Furthermore, most commercial GPRs operate in pulsed mode in which the wavelength is fixed and frequency varies with surrounding permittivity, as opposed to surface acoustic wave transducers operating at a fixed frequency range defined by the spatial period of electrodes patterned on a piezoelectric substrate. Knowing these limitations, mitigation strategies include replacing the drifting analog timebase generator with a stable digital solution and using a wideband antenna in addition to deploying sensors in environment with stable permittivity such as concrete, so that the antenna operating frequency remains within the transducer passband. This prospective study must now be completed with a practical deployment scenario in which the benefit of the embedded sensor complementing the non-destructive GPR characterization is to be addressed.

## 8. Perspectives

We emphasize that pulsed-mode GPR is one usage case that seems most favorable to the deployment of acoustic transducers as cooperative targets. Another class of industrial RADAR are the microwave FMCW (Frequency-Modulated Continuous Wave) RADAR systems deployed for measuring the height of fluids and granular media. The resolution of these devices is, as for all RADAR systems, given by the inverse of their bandwidth, which is more easily maximized at higher carrier frequency. Hence, industrial water level FMCW RADAR systems typically operate in the tens of GHz range, well beyond the reach of acoustic transducers. The general philosophy described here is nevertheless applicable, when replacing the acoustic transducer with a dielectric transducer, made compact by including multiple reflections between boundary conditions, e.g., as found in Fabry–Perot cavities or more commonly in dielectric resonators. 

## Abbreviations

The following abbreviations are used in this manuscript:

DAC	Digital to Analog Converter
FDTD	Finite Difference Time Domain
FMCW	Frequency-Modulated Continuous Wave
FPGA	Field Programmable Gate Array
FSPL	Free Space Propagation Loss
GPR	Ground Penetrating RADAR
IDT	Inter-Digitated Transducer
PLL	Phase Locked Loop
ppm	parts per million
PRR	Pulse Repetition Rate
PVC	Poly(Vinyl Chloride)
RADAR	RAdio Detection And Ranging (here, “ranging” is a measurement of the quantity sensed by the target)
RFID	Radio-Frequency IDentification
SAW	Surface Acoustic Wave

## References

[1] Kofman L., Ronen A., Frydman S. (2006). Detection of model voids by identifying reverberation phenomena in GPR records. J. Appl. Geophys..

[2] Brewster M.L., Annan A.P. (1994). Ground-penetrating radar monitoring of a controlled DNAPL release: 200 MHz radar. Geophysics.

[3] Saintenoy A., Hopmans J.W. (2011). Ground penetrating radar: Water table detection sensitivity to soil water retention properties. IEEE J. Sel. Top. Appl. Earth Obs. Remote Sens..

[4] Annan A., Davis J. (1976). Impulse radar sounding in permafrost. Radio Sci..

[5] Laurens S., Balayssac J., Rhazi J., Klysz G., Arliguie G. (2005). Non-destructive evaluation of concrete moisture by GPR: Experimental study and direct modeling. Mater. Struct..

[6] Lachowicz J., Rucka M. Experimental and numerical investigations for GPR evaluation of reinforced concrete footbridge. Proceedings of the 2016 16th International Conference on Ground Penetrating Radar (GPR).

[7] Stoney R., Geraghty D., O’Donnell G.E. (2014). Characterization of differentially measured strain using passive wireless surface acoustic wave (SAW) strain sensors. IEEE Sens. J..

[8] Bruckner G., Stelzer A., Maurer L., Biniasch J., Reindl L., Teichmann R., Hauser R. A high-temperature stable SAW identification tag for a pressure sensor and a low-cost interrogation unit. Proceedings of the 11th International Sensor Congress (SENSOR).

[9] Scherr H., Scholl G., Seifert F., Weige R. Quartz pressure sensor based on SAW reflective delay line. Proceedings of the 1996 IEEE Ultrasonics Symposium.

[10] Beckley J., Kalinin V., Lee M., Voliansky K. Non-contact torque senseor based on SAW resonators. Proceedings of the IEEE International Frequency Control Symposium and PDA Exhibition.

[11] Wand W., Lim C., Lee L., Yang S. (2009). Wireless surface acoustic wave chemical sensor for simultaneous measurement of CO_2_ and humidity. J. Micro/Nanolithogr. MEMS MOEMS.

[12] Shibata H. (1983). What research is needed in reliability and failure prevention in the field of anti-earthquake design of industrial facilities. J. Vib. Acoust. Stress Reliab. Des..

[13] Suppasri A., Mas E., Charvet I., Gunasekera R., Imai K., Fukutani Y., Abe Y., Imamura F. (2013). Building damage characteristics based on surveyed data and fragility curves of the 2011 Great East Japan tsunami. Nat. Hazards.

[14] Charvet I., Macabuag J., Rossetto T. (2017). Estimating Tsunami-Induced Building Damage through Fragility Functions: Critical Review and Research Needs. Front. Built Environ..

[15] Chen J.H., Su M.C., Chen C.Y., Lin S.C. (2014). Developing a damage assessment model for bridge surroundings: A study of the disaster caused by Typhoon Morakot in Taiwan. Civ. Eng. Environ. Syst..

[16] Dong L., Shan J. (2013). A comprehensive review of earthquake-induced building damage detection with remote sensing techniques. ISPRS J. Photogramm. Remote Sens..

[17] Kurata N., Spencer B.F., Ruiz-Sandoval M. Building risk monitoring using wireless sensor network. Proceedings of the 13th World Conference on Earthquake Engineering.

[18] Stockman H. (1948). Communication by means of reflected power. Proc. IRE.

[19] Preradovic S., Karmakar N.C. (2010). Chipless RFID: Bar code of the future. IEEE Microw. Mag..

[20] Zhang J., Tian G.Y., Marindra A.M., Sunny A.I., Zhao A.B. (2017). A review of passive RFID tag antenna-based sensors and systems for structural health monitoring applications. Sensors.

[21] Thomson D., Card D., Bridges G. (2009). RF cavity passive wireless sensors with time-domain gating-based interrogation for SHM of civil structures. IEEE Sens. J..

[22] Zhao Y., Li Y., Pan B., Kim S.H., Liu Z., Tentzeris M.M., Papapolymerou J., Allen M.G. (2010). RF evanescent-mode cavity resonator for passive wireless sensor applications. Sens. Actuators A Phys..

[23] Kubina B., Schusler M., Mandel C., Mehmood A., Jakoby R. Wireless high-temperature sensing with a chipless tag based on a dielectric resonator antenna. Proceedings of the 2013 IEEE SENSORS.

[24] Morgan D. (2007). Surface Acoustic Wave Filters.

[25] Hashimoto K.Y. (2000). Surface Acoustic Wave Devices in Telecommunications.

[26] Royer D., Dieulesaint E. (1999). Elastic Waves in Solids II: Generation, Acousto-Optic Interaction, Applications.

[27] Bao X.Q., Burkhard W., Varadan V.V., Varadan V.K. SAW temperature sensor and remote reading system. Proceedings of the IEEE Ultrasonics Symposium.

[28] Pohl A., Seifert F., Reind L., Scholl G., Ostertag T., Pietsch W. Radio signals for SAW ID tags and sensors in strong electromagnetic interference. Proceedings of the IEEE Ultrasonics Symposium.

[29] Pohl A., Reindl L. (1998). Measurement of physical parameters of car tires using passive SAW sensors. Advanced Microsystems for Automotive Applications 98.

[30] Reindl L. (2004). Wireless passive SAW identification marks and sensors. Functional Micro- and Nanosystems.

[31] Bulst W.E., Fischerauer G., Reindl L. (2001). State of the art in wireless sensing with surface acoustic waves. IEEE Trans. Ind. Electron..

[32] Scholl G., Schmidt F., Ostertag T., Reindl L., Scherr H., Wolff U. Wireless passive SAW sensor systems for industrial and domestic applications. Proceedings of the 1998 IEEE International Frequency Control Symposium.

[33] Gizeli E. (2003). Acoustic transducers. Biomolecular Sensors.

[34] Charvat G.L. (2014). Small and Short-Range Radar Systems.

[35] Allen C.T., Shi K., Plumb R.G. (1998). The use of ground-penetrating radar with a cooperative target. IEEE Trans. Geosci. Remote Sens..

[36] Maierhofer C., Reinhardt H.W., Dobmann G. (2010). Non-Destructive Evaluation of Reinforced Concrete Structures: Non-Destructive Testing Methods.

[37] Plessky V.P., Reindl L.M. (2010). Review on SAW RFID tags. IEEE Trans. Ultrason. Ferroelectr. Freq. Control.

[38] Friedt J.-M. ProEx GPR Control web site. https://sourceforge.net/projects/proexgprcontrol/.

[39] Daniels D. (2004). Ground Penetrating Radar (IEE Radar, Sonar, Navigation and Avionics Series).

[40] King R. (1986). Antennas in material media near boundaries with application to communication and geophysical exploration, Part I: The Bare Metal Dipole. IEEE Trans. Antennas Propag..

[41] Kurokawa K. (1965). Power waves and the scattering matrix. IEEE Trans. Microw. Theory Tech..

[42] Loo C.H., Elmahgoub K., Yang F., Elsherbeni A.Z., Kajfez D., Kishk A.A., Elsherbeni T., Ukkonen L., Sydanheimo L., Kivikoski M. (2008). Chip impedance matching for UHF RFID tag antenna design. Prog. Electromagn. Res..

[43] Tang T.G., Tieng Q.M., Gunn M.W. (1993). Equivalent circuit of a dipole antenna using frequency-independent lumped elements. IEEE Trans. Antennas Propag..

[44] Schelkunoff S.A., Friis H.T. (1952). Antennas: Theory and Practice.

[45] Warren C., Giannopoulos A., Giannakis I. (2016). gprMax: Open source software to simulate electromagnetic wave propagation for ground penetrating radar. Comput. Phys. Commun..

[46] Davis J.L., Annan A. (1989). Ground-penetrating radar for high-resolution mapping of soil and rock stratigraphy. Geophys. Prospect..

[47] Ihamouten A., Villain G., Dérobert X. (2012). Complex permittivity frequency variations from multioffset GPR data: Hydraulic concrete characterization. IEEE Trans. Instrum. Meas..

[48] Annan A., Waller W., Strangway D.W., Rossiter J.R., Redman J., Watts R. (1975). The electromagnetic response of a low-loss, 2-layer, dielectric earth for horizontal electric dipole excitation. Geophysics.

[49] Friedt J.M. (2017). Passive cooperative targets for subsurface physical and chemical measurements: A systems perspective. IEEE Geosci. Remote Sens. Lett..

[50] Johansson B.A. (2002). Ground Penetrating Radar Array and Timing Circuit. U.S. Patent.

